# The Influence of Two Teaching Approaches on Foot Loading in Skiing Beginners—A Comparative Study

**DOI:** 10.3390/s24237653

**Published:** 2024-11-29

**Authors:** Nicolas Kurpiers, Luca Gersmann, Kai Reinhart, Nils Eden, Uwe G. Kersting

**Affiliations:** 1 Department of Sport Science, University of Hildesheim, 31141 Hildesheim, Germany; nilseden@icloud.com; 2 Department of Sport Science, University of Münster, 48149 Münster, Germany; luca.gersmann@icloud.com (L.G.); reinkai@uni-muenster.de (K.R.); 3 Institute of Biomechanics and Orthopaedics, German Sport University Cologne, 50933 Cologne, Germany; u.kersting@dshs-koeln.de

**Keywords:** alpine skiing, load distribution, loadsole, snowplow, one-ski-method, teaching approach

## Abstract

(1) Background: Alpine skiing, with its long history, has experienced numerous innovations and developments on all levels ranging from technology to fashion over the past 120 years. However, teaching approaches for beginners remained quite consistent for many decades and are mainly grounded in experience. The One-Ski-Method (OSM) is an alternative approach to the predominant snowplow (SP) method with the strategy to initially experience and acquire the elementary positions and actions on one ski in order to subsequently transfer these to two skis. The aim of the study was to compare the effects of the SP and the OSM by assessing the position of the ski via load distribution sensors. (2) Methods: A total of 33 participants were groupwise randomly assigned to the two methods and tested via load insoles on the first and the fifth day on a moderate slope for six turns. Between the two measurements, the groups were instructed according to the SP or the OSM methods, respectively. The data were analyzed via Matlab and SPSS. (3) Results: The OSM group showed a significantly greater forefoot load than the SP group (*p* = 0.029). The SP group developed a greater rearfoot loading from pre- to post testing. (4) Conclusions: The findings make it perceivable that OSM learners acquire a beneficial specific position on the ski due to the exercises of the OSM.

## 1. Introduction

Alpine skiing is a popular sport offering health benefits [1] and outdoor experience [2] over the whole life span. Children can easily learn how to ski from the age of three in a Ski-Kindergarten while even the elderly can still perform turns in the snow provided they remain healthy. Also for rehabilitation purposes, skiing can be an attractive option to find the path back to normality [3]. The history of skiing has received a certain dynamic, e.g., regarding the equipment but maybe also fashion [4]. In the 1960s, the ‘modern’ ski boot was invented with a higher shaft, and later in the 1990s, the waisted carving ski was released which remains the main ski design until today and enables carved turns on the edge with a comparably short radius. Other innovations came and disappeared such as the Monoski in the 1980s or the Soft Boot in the 1990s. Also in the theory of alpine skiing and the teaching philosophy and the history of the guidelines (in Germany), some aspects were temporarily more emphasized or neglected subsequently such as the ‘high-unloading’ or different competencies of ski instructors such as communicating, motivating, instructing, etc. [5]. There were also guidelines without real innovations, but only slight changes in terminology or structure regarding illustrations and explanations [6]. However, within more than 120 years of skiing instructions in alpine skiing the concern about, and development of, teaching methods in alpine skiing was never of a particular focus. For a variety of positive aspects in alpine skiing, such as the experience of gliding and turning, the way how to learn skiing, i.e., the method plays a crucial role in a successful movement experience. What a beginner experiences and feels, and how a beginner can adopt motion patterns is highly dependent on the learning path and the methodical exercises.

According to Ericsson [7], once a motion pattern is automated and thus represented in the motor cortex, the movement is no longer consciously controlled, which in turn makes conscious changes in the execution of the movement more difficult. Thus, the teaching method is likely highly important for the acquisition of motion patterns and their consolidation in motor learning, and therefore, underlying movement elements should be as similar as possible to the final technique goal. It has been observed in the past that the snowplow can potentially lead to a backward lean position which may become a problem once this position is acquired. In the late 1950s, the authors of the German guidelines had already expressed the view that they did not intend to continue teaching based on the snowplow and that they took it as a necessary evil [8]. Going forward in time, in the guideline from 2019, it is stated that the detrimental effects of the snowplow are an awkward position of the femoral head in the acetabulum and that the position is exhausting and can lead to pain or cramps [6].

### 1.1. Teaching Approaches in Alpine Skiing

Assuming that parallel skiing is the general goal of all the teaching paths as all terrains require a parallel ski position for the capacity to act appropriately, we can differentiate between tools and methods. One tool was suggested by Maegerlein et al. [9] using short skis. The so-called ‘Short Ski Method’ follows the idea of learning how to ski with increasing ski lengths. A very short ski is certainly easier to turn and carries less risk while the basic actions are similar. It needs to be noted that this approach can indeed lead to the goal of ‘parallel skiing’; however, first, it is quite costly in terms of equipment, and second, it is questionable if it actually can be named as a method as a short ski is first of all a methodical tool and does not include any specific teaching concept or methodological structure.

**Figure 1:**
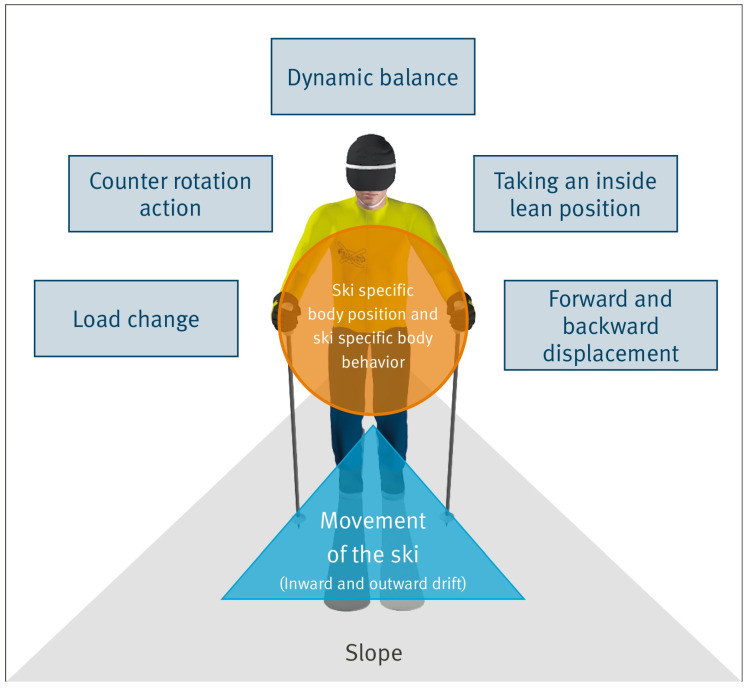
Illustration of the relevant elements for a turn in alpine skiing [10].

The presently predominant teaching way, the ‘snowplow’ (SP) was originally based on quite plausible aims, specifically mastering an ungroomed slope with rather simple equipment. Further beneficial aspects are the opportunity of braking at slow speeds, speed control in narrow passages, and the feeling of safety in straight runs due to stability on a wider base of support. However, there is a great difference between using the snowplow for braking (e.g., before queuing at a lift), for control in a slow straight run, or in a methodical way in order to learn how to turn. As a methodical approach, we appear to observe a few quite counter-productive side effects. Those may outweigh the supposed benefits because elementary aspects and motion patterns for parallel skiing (see Figure 1) are not being acquired by using the snowplow as the basic element of the skiing technique.

Both inside edges are permanently loaded in the snowplow position, and thus there is no edge change possible within a turn. The edge change, however, belongs to the elementary motion components in parallel alpine skiing. In this way, the sensitivity for the edge change and also the inside ski cannot be experienced which is important, even though the outside ski is commonly higher loaded. Further, in the snowplow, the skier leans towards the outside of the turn, whereas in parallel skiing, an inside lean is required and functional while a pelvis–trunk angle in the frontal plane may be used to control the inside lean and edging of the skis. Therefore, parallel skiing can be characterized by playing with the balance and imbalance of positions and actions which does not happen in the snowplow due to a quite stable position above a wide base of support. Hence, it appears obvious that the elementary actions of parallel skiing are not required and are, therefore, not intentionally taught through the snowplow. This is the reason why it was termed an ‘indirect approach’ [11] as the acquired motion patterns need to be unlearned subsequently for the transfer to parallel skiing.

We can summarize that the snowplow as a methodical approach has a long tradition and works in a way, however, in a rather indirect way. We have asked the question of whether the snowplow is actually a method or whether it is just a deviant but still the main skill or technique variant used in most skiing guidelines, with which they center on or build on as a fundamental skill. The alternate question is then which technically relevant actions are consequently required in skiing turns that could then serve as a base for a direct teaching path to parallel skiing from the start.

The *’One-Ski-Method’* (OSM) [10] is based on biomechanical research results [12,13,14] which led to the determination of elementary and relevant body actions which are the minimum motions which are required to turn the ski on the slope, specifically the following:

- Taking an inside lean position;
- Load Change;
- Counter Rotation Action;
- Forward and Backward Displacement of the Body (see Figure 1).

Additional requirements to control turns are a specific body position, dynamic balance, and height adjustments. In parallel alpine skiing, however, there is a whole variety of accentuations of these actions for different situations, respectively, e.g., off-piste, varying snow conditions, varying slope characteristics, etc.

The One-Ski-Method was derived from fundamental technique understanding based on these findings. The core of the resulting teaching method is to practice just or predominantly the relevant actions in a very learning-goal-oriented manner. The strategy can be summarized by learning the ski-specific positions and actions through various exercises first on just one ski (left and right) in order to gradually transfer the learned actions to two skis.

### 1.2. Scientific Approaches

There are study-based pointers that the OSM might be an appropriate method for novices for learning faster [15]. Regarding the investigation of the position on the ski from a biomechanical point of view, there are various options such as full-body suits with sensors, different video analysis systems, mobile force plates, or load-sensitive insoles. The latter is an approach conceivably easier to apply in terms of equipment efforts and is often used in combination with a conventional video camera in order to be able to synchronize the pressure values with the respective movement phases. The pressure distribution underneath the feet cannot directly serve as a measure for the position on the ski; however, it is feasible to derive hints as to whether the position of the skier is beneficial concerning control (mid- to front lean) or rather detrimental and even potentially imperiling (back lean). Besides the benefits of the ability to act appropriately and regulate the turns and adapt to various slope conditions, a controlled position additionally serves as a safety measure. A back lean position was found to be the main reason for most knee injury mechanisms and thus needs to be avoided [16,17,18].

Quite a few research groups utilized different pressure insole systems in order to validate it [19] or compare its precision to another system, e.g., force sensors [20], or to find out about regulatory mechanisms while turning and the most beneficial load distribution during turns mostly on expert or elite skiers. The application of such measurement to beginners has not yet been documented.

The aim of the current study was to compare the snowplow as a teaching method with the One-Ski-Method regarding differences in the load distribution as an indicator for (a) a beneficial position on the ski for turn execution and a good prerequisite for successful motor learning and (b) for control of the skis. It was hypothesized that through the OSM, a beneficial position (mid- to forward lean tendency) will be acquired from the start of the learning process due to repetitive practice in goal-oriented exercises and methodical tools (see the Section 2).

## 2. Materials and Methods

### 2.1. Sample

Participants were recruited during the skiing excursions of two different German Universities and one high school (N = 33; 17–27 years). They were excursionwise randomized and divided into two intervention groups which comprised 17 (SP: 9 males, 8 females; 21 ± 3.4 years; 68 ± 9.5 kg) and 16 persons (OSM: 9 males, 7 females; 22 ± 2.3 years; 72.8 ± 9.7 kg). All the participants were beginners and started with half a day of ski lessons in order to be able to perform the test procedure for the first time (t1). After five days of skiing instructions, they were tested for the second time (t2). All the participants signed informed consent prior to the test runs. The investigation was conducted in accordance with the Declaration of Helsinki. Lacking previous studies on this approach, pilot tests on two groups of six beginners each being taught after the two teaching methods, respectively, were used to estimate the sample size. Effect sizes were transformed to fit the F-statistic, comparing two groups pre and post. The effect size of 0.33 resulted in N = 24 (12/12) based on a low (r = 0.35) correlation between pre- and post-measures, an alpha level of 0.05, and a power of 0.8 (G-Power 3.1.9.6, Heinrich-Heine-University, Düsseldorf, Germany).

### 2.2. Test Sessions

The test sessions consisted of three runs on a moderate slope of approximately 18° inclination. Prior to the test runs, pressure insoles of the brands Novel (Loadsol, 100 Hz frequency) and Moticon (ReGo Sensor Insoles, 50 Hz frequency) were placed inside the ski boots bilaterally. The soles were operated for Novel via the app loadsol, using an iPhone 11 (Apple Inc., Chennai, India). The soles of Moticon were operated via the app OpenGo using a tablet Samsung Galaxy Tab A (Samsung Electronics, Thai Nguyen, Vietnam). The soles were battery-powered. In addition to the load distribution measurements, all the participants were recorded using a 2015 iPad Air (Apple Inc., Shenzhen, China) using the app Timestamp Camera (Apple Inc.) to synchronize measurement and movie recording time offline after the tests. This video served as a visual aid for the data analysis. For Novel, the smartphone was fixed on the front and center of the body to ensure a good transmission rate (Bluetooth). Moticon stored the data on internal memory (no Bluetooth necessary).

Prior to the test run, the participants were instructed to try to meet the criteria of fluent, moderate speed, and moderate turn frequency within the marked corridor of approximately five meters. Immediately at the beginning of the test run, the participants stamped their right foot once to detect an obvious peak in the force signal for the later evaluations and synchronization of the video when using the novel insoles. All the participants were instructed to ski to the lift at the end of their measurement slope to return to the beginning of the test area. During the lift ride, the measurement system was prepared for the next participant (2 sole pairs were available).

### 2.3. Data Analysis

The exported data of the pressure measurement soles were analyzed in Matlab (MatLab 2021b, TheMathWorks, Nattick, MA, USA). In the case of the loadsol data (N = 14 SP, 12 OSM), the force curves of the sensor surfaces were read in for the entire test and filtered with a Butterworth filter (lowpass, 20 Hz, 4th order). In the case of the Moticon soles (N = 3 SP, 4 OSM), the pressure values of the individual sensor types were read in, multiplied by the respective sensor area, and subsequently summed up so that the forefoot and rearfoot areas matched the geometry of the loadsol soles as closely as possible. The same filter as for the loadsol data was applied to the raw sensor data. Afterward, the total forces for right and left were plotted graphically for the entire measurement period, and with reference to the log videos, six turn cycles were manually selected. A crossing point of the right and left total forces, i.e., load shift between skis, defined the beginning of one cycle. Care was taken to select the cycle from a time interval where a clear rhythmical and consistent force profile was depictable. Subsequently, the individual turns were also separated manually and the mean and maximum force values for each individual turn were determined and saved in a tabular format.

A statistical analysis was performed using SPSS (Version. 28.0 for Mac, SPSS Inc., Chicago, IL, USA) and Microsoft Excel (Version 16.42). The means and standard deviations of the dependent variable (OSM vs. SP) were calculated for all the participants. The groups were tested for normal distribution. In the case of normally distributed data, the *t*-test for dependent samples was applied. Otherwise, non-parametric tests (Mann–Whitney-U-Test and Wilcoxon Test) were chosen. Statistical significance was set at *p* < 0.05. Effect sizes were classified according to Cohen (1992) as weak effect (r = 0.10), medium effect (r = 0.30), and strong effect (r = 0.50).

### 2.4. Intervention

The teaching way via snowplow is common and presumably well known. Otherwise, all the pertinent guidelines of the associations provide the respective methodical exercises and steps. Details about the One-Ski-Method can be gained via the web page www.ein-ski-methodik.de (accessed on 25 September 2024), the latest release of the book [10], and three different articles [11,15,21]. The centerpiece of that teaching path will briefly be introduced in the following.

**Figure 2:**
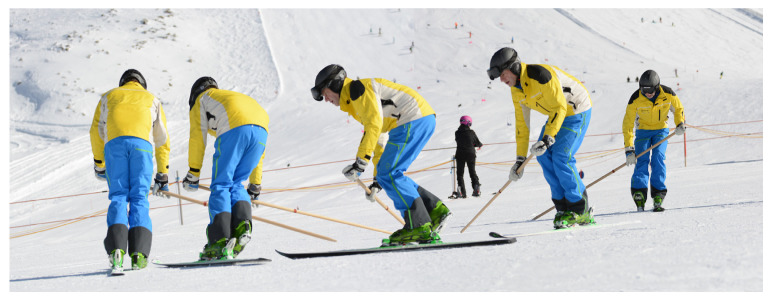
A long rod as methodical tool (here, turn over the fall line on the left outside ski) [10].

The essential methodical aid is a long wooden rod of 2 m length (Figure 2). It slides on the inside of the turn and serves as a psychological and mechanical support for beginners as they are usually less inhibited from leaning into the curve, and it promotes a controlled inside position. It can be considered as an assistive tool for the phase when balancing capacity is not yet fully developed, but using it the inside lean and canting changes can be practiced. With a minimum of basic exercises (commonly one half-day), they can progress to parallel skiing with two skis and the rod, which usually means a great success for beginners. In a very reduced illustration, the main components of the OSM are a variety of gliding exercises on one ski, always to both the inside and the outside edges. Repetitive turns around a mark in the flat on one consciously edged ski belong to the basic drills and a pool of variations in a turn on one ski with the rod on a moderate slope inclination. A regular change from drills on the left leg to the right leg and back is mandatory. These variations imply single turns with and without crossing the fall line. Subsequently, there is the option to perform these variations on the inside ski too, and/or progress to the same exercises with two skis.

In a previous study, the exhaustion of the ski lessons was rated moderate which is in line with the measured heart rates during ski lessons [22].

## 3. Results

The Kolmogorov–Smirnov test showed that the measures of the variables SP-Pre (*p* = 0.86, N = 17), SP-Post (*p* = 0.89, N = 17), OSM-Pre (*p* = 0.46, N = 16), and OSM-Post (*p* = 0.64, N = 16) were all normally distributed. Therefore, a one-factor repeated measures ANOVA was employed to assess group differences and compare the pre–post alterations within each group using the rearfoot–forefoot ratio as the main outcome.

The One-Ski-Method group (Mean ratio = 1.75) showed a relatively lower forefoot load at t1, i.e., a larger rearfoot–forefoot ratio, than the snowplow group (mean ratio = 1.46) while this difference was not significant (*p* = 0.095).

**Figure 3:**
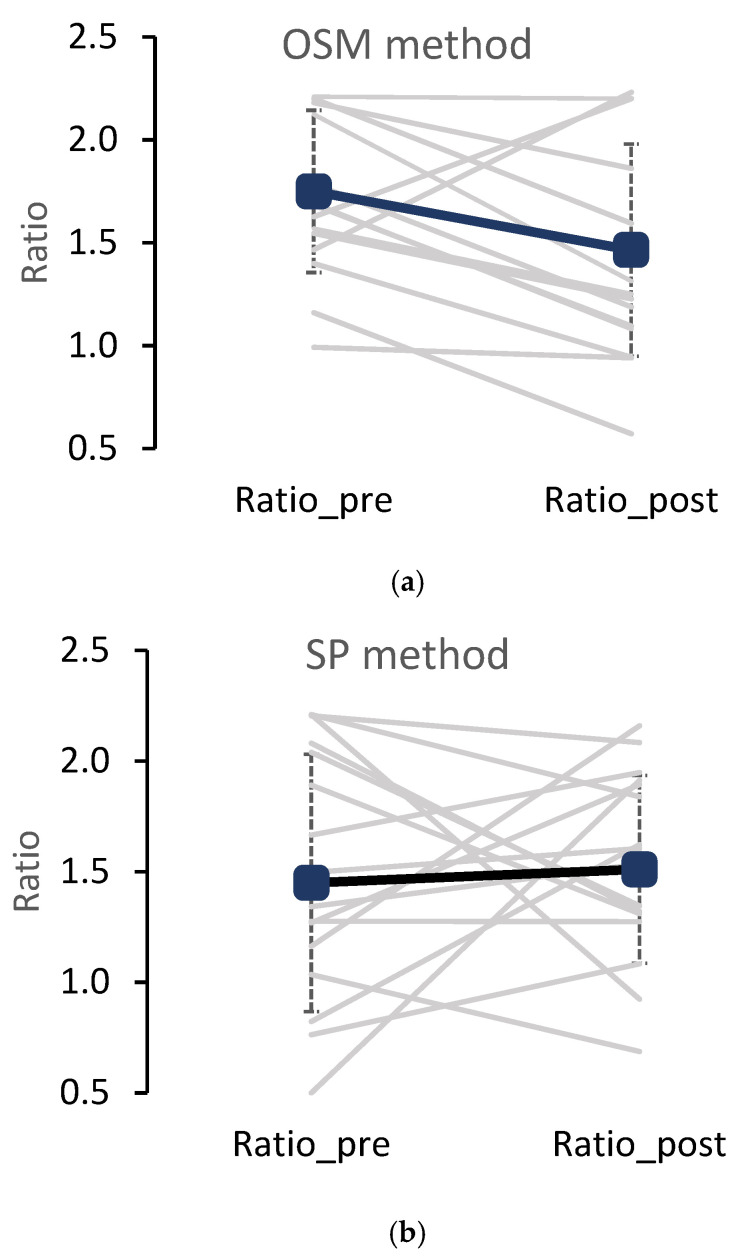
Mean rearfoot–forefoot ratio for measurement time points (pre) and (post) for (**a**) OSM method (N = 16) and (**b**) SP method (N = 17).

The results demonstrated that the OSM had a significant effect on rearfoot–forefoot distribution (Figure 3). The difference was −0.29 from pre- to post test (*p* = 0.0178). The effect size according to Cohen (1992) was r = 1.09 and thus corresponds to a strong effect.

The SP method had no significant effect on the rearfoot–forefoot ratio (*p* = 0.772) with the mean value slightly increasing from pre- to post test (Table 1).

## 4. Discussion

The main objective of this study was to compare two teaching approaches regarding their effects on the posture on the ski. These effects were assessed by the measurement of the load distribution of alpine skiing beginners underneath their feet referring to an anterior–posterior load shift. The snowplow and the One-Ski-Method were compared and the measurement times were on the first day (t1) and on the fifth day (t2). The results demonstrated that the OSM beginners showed a consistent shift and a significantly reduced ratio, representing an anterior load distribution after five days of skiing compared to the SP beginners with a large effect size (r = 1.09).

The OSM learners and SP learners started off with a similar load distribution. However, the SP and OSM learners showed an opposed effect over the course of the 5 days, resulting in a virtually unchanged backward lean position after five days of skiing (t2) for the SP group. Additionally, the development of the SP participants was much more variable with changes going in both directions (Figure 3). Beginners often show a tendency for a more upright and slightly backward leaning position. This position seems to be unchanged when the historically evolved teaching approach is used. This development indicates that the SP tradition seems to discourage a development away from a disadvantageous body orientation over the ski. This is presumably enforced by the stemming movement on the outside ski as the edge pressure of the outside ski is increased by this action and could pull the skier backward due to the decentralized mounting of the binding.

For the OSM group, a directed development was observed. This group started off from a slightly higher but not substantially different base line and developed an increasing load on the forefoot area accompanied by a more anteriorly directed rearfoot–forefoot ratio. This trend is very consistent compared to the SP group (Figure 3). The increasing forward loading of the OSM learners was likely an effect of the teaching approach as one-ski-turns are easier to perform with a clear front lean which was continuously reinforced and may even lead towards an automatized motion pattern. It can be assumed that OSM learners acquire a beneficial skiing-specific position on the ski from the start of the learning process. This would match various observations over the years, i.e., the consistent mid- to forward lean positions of novices from the start. Further, it could explain that no great differences in magnitude were developing from t1 to t2 while the improvement was significant for the group and in a forward direction in 14 out of 16 skiers.

It is likely that the OSM training leads to such a beneficial stance which in turn facilitates the control of the above-named elementary actions. For the subsequent technique development in regard to edging, rhythm, position, or varying radii, beginners will have a good prerequisite for mastering challenging skiing situations and environments later on in their careers leading to improved mobility in the knees in a slightly deeper position and an improved readiness for the adjustments in six degrees of freedom. Such situations and environments could imply absorbing uneven surfaces, or moguls; mastering deep snow; steep slopes; or just frequent short turns.

In a previous study, SP and OSM learners were assessed using video footage only in order to rank selected skiing abilities on the first and the fifth day of instructions. Expert ski instructors ranked the turns from 1 (very poor) to 10 (excellent) based on the following criteria: skis parallel, position on the ski (forward lean position), canting, tight upper body, pole planting, tilting the knees, and smooth/fluent turns. In that study, four out of seven items were ranked significantly better for the OSM group [15]. This is in accordance with a potentially more beneficial position corroborated in the current study.

The acquired position with more anterior load distribution may also be beneficial regarding knee injury prevention. The knee is the most common site of injury, with reports ranging from 27% to 41% of all alpine skiing injuries [23]. Most known knee injury mechanisms result from a backward seated position and lead to a loss of control promoting accidents and increasing the load on the ligamentous structures of the knee joint. Three mechanisms have been quoted in the context of knee injuries in skiing: (a) boot-induced anterior drawer (BIAD), (b) phantom foot phenomenon, and (c) the valgus external rotation [24]. With a generally less backward-oriented position, skiers may remain in control of balance and edge loading and they may be able to better avoid potentially harmful movements. Thus, the teaching approach can potentially contribute as an injury prevention strategy in that it aids in avoiding critical body positions by automatizing a beneficial position with regard to the anterior–posterior shift in the load distribution.

In terms of motor learning, Thomson and Wolpaw [25] argued that local physiological adaptations in the nervous system lead to broader beneficial changes in the central nervous system with widespread adaptive changes which lead to an expanded motor action repertoire by providing the opportunity for a new ‘negotiated equilibrium’ within the motor control system leading to improvements in learning without compromising already known motor solutions. Further, it has specifically been reviewed that the developments of sensory systems and motor systems are reciprocally linked [26] and that a coupled development of both systems is especially advantageous for motor learning. Taken together, these basic neuro-physiological mechanisms make it perceivable that the practice of biomechanically beneficial motor actions from the early initiation of a motor learning process will lead to faster and functional adaptations. On the contrary, the provision of contradictory sensory perceptions and instructions may be particularly misleading to a novice when learning a new motor skill.

Expanding on the described fundamental physiological processes, neuropsychologic learning theories propose that after having acquired a new skill, i.e., the snowplow in skiing as a contextual motion solution, an engram for this specific movement pattern has been created in the motor cortex [27]. Subsequently, it becomes more challenging to alter such an engram in a similar context, or to trigger a different motor action and save this as a new pattern in a new engram, or alter or replace an existing one. The initial, originally saved pattern would always be triggered first and make it harder for the novice to apply the key movements as required.

Based on this, the solution should be to methodically bypass the unconscious retrieval of misleading motor programs by modifying the task or the situation that triggers the retrieval. In the current application, this could be managed by introducing the initial exercises on one ski which makes it impossible to stem or use the snowplow, or alternatively, to use the long rod with two skis which would make the snowplow unnecessary.

We have to ask the question of how such scientific findings can be transferred and implemented into teaching practice. Moreover, it may be questioned whether the efficiency of the skiing associations’ curricula, particularly for beginners, gets reflected on and evaluated whatsoever. The problem, however, lies in an earlier step, i.e., the technique understanding that a teaching approach should be based on. The snowplow, as the predominant teaching path, has a long history. As is known from other areas, such traditions are not easy to leave behind. However, this approach is indeed experience-based as most of the skiers learned with that method, but it is not evidence-based. First of all, there is a discrepancy regarding the technique understanding and the mechanisms of turning. The associations assume leg twisting and high-unweighting as relevant mechanisms for turns, whereas the OSM is also based on the argument that we cannot twist the legs with the skis attached ourselves, but we need to create a torque between slope and ski in order to turn the whole ski–skier system. Further, a vertical unweighting movement is not functional for turns as we need the weight on the edges in order to maintain the dynamic balance. Thus, the motion technique needs to be clear as the goal for a methodical approach. Afterward, we can either develop a path or we are able to evaluate an existing one. The inventors of the One-Ski-Method carried that out whereas skiing associations stagnated in that regard. In order to establish the One-Ski-Method as a methodical approach a common denominator is required with respect to technical aspects, body actions, and positions, and also the detrimental effects of the snowplow that were already known in the German guidelines of the 1950s [8].

The arguments presented above lead to the suggestion that we should simply offer a setting for the learners that promote the necessary experiences, the needed elementary body actions, and positions to solve the task in a goal-oriented manner. The automatization as a change in neuronal networks is a physiological process in which we cannot directly intervene. However, we can support the learning process by providing favorable conditions such as moderate slopes, reasonably selected exercises, accentuations, and specific facilitations, e.g., initial exercises on one ski, the rod as a supportive tool, or a safe atmosphere to avoid anxiety.

Loadsol sensors, as a measurement device as utilized in this study, can also be used as a real-time feedback system. It is possible to record a video and the load data synchronized in order to directly show it to the skier after a run. Such systems have previously been introduced for advanced or competitive skiers (e.g., https://getcarv.com); however, it could be worth investigating whether and how it could be applicable and possibly beneficial for learners. It could be hypothesized that novices would possibly adapt to an appropriate position on the ski or be reminded of the ski-specific body behavior (see Figure 1) in the course of the first skiing days.

As a limitation of the study, it needs to be noted that we utilized two different measurement devices, specifically the Novel system and the Moticon system, for the assessment of load distribution. We aimed at a comparable split between forefoot and rearfoot which cannot be warranted due to the different sensor geometries. This situation evolved due to one of the systems beginning to show technical failure when used in a ski boot. This led to the elimination of some measurements while the remaining trials were carefully checked to maintain a sufficient sample size. Carrying out such measurements on absolute beginners who act in a new and challenging environment which is likely different between testing days bears the limitation that anxiety or motivational aspects were hard to control and may have led to a greater variability. Generally, the measurement of load distribution is just an indicator of the posture; however, the link between load distribution and stance has been shown several times previously [28,29,30]. Another limitation is the fact that the whole reaction force cannot be represented by pressure insoles as a part of the body weight gets absorbed by the ski boot shaft. However, an Austrian research group rated this limitation reasonable [31].

## 5. Conclusions

In conclusion, the teaching method has an influence on foot loading in alpine skiing that is likely to be caused by teaching exercises and the respective body sctions. Foot loading is an indicator of forward lean which is in turn a central component in alpine skiing.

The OSM training carries the potential to reach an appropriate position on the ski which is important for (a) an efficient acquisition of the motion patterns and (b) for safety reasons. Thus, beginners can prospectively learn ski-specific behavior and body actions quicker, and concurrently, the predominantly high number of knee injuries in alpine skiing could be reduced. Whether this is indeed the case could be the objective of further studies. The application of loadsols for direct feedback in beginners could be an action to be investigated soon.

Further research is warranted that, first of all, determines the relevant body actions needed for turn initiations. Afterward, we should conduct further studies that evaluate the efficiency of the goal orientation of different approaches, such as the snowplow.

## Figures and Tables

**Table 1:** Mean rearfoot–forefoot ratio for measurement time points (pre) and (post) for (a) OSM method (N = 16) and (b) SP method (N = 17). The compared conditions include different groups (OSM 1, 2; SP 1, 2) which were tested at different skiing locations.

	N	Pre		Post		Sig.
		Mean	(SD)	Mean	(SD)	
OSM_1	8	1.80	(0.37)	1.53	(0.59)	
OSM_2	8	1.70	(0.44)	1.40	(0.46)	
OSM	16	1.75	(0.39)	1.46	(0.52)	*p* = 0.0178
SP_1	7	1.53	(0.70)	1.55	(0.45)	
SP_2	10	1.38	(0.48)	1.47	(0.42)	
SP	17	1.46	(0.58)	1.51	(0.42)	*p* = 0.722

## Data Availability

Data access is restricted according to the Institutional Review Board to protect confidential or proprietary information. We will make the data available upon request, with permission for the purposes of peer review.
